# Plant–Atmosphere Interactions in Urban Vineyards of Rome: A Digital Phenotyping Approach for the Functional Selection of Grapevine Cultivars

**DOI:** 10.3390/plants15193014

**Published:** 2026-09-30

**Authors:** Elena Brunori, Alessandro Rocci, Ilenia Baraccani, Elisa Ovidi, Chiara Anselmi, Dania Valli, Carlo Calfapietra, Fabio Attorre, Rita Biasi

**Affiliations:** 1 Department for Innovation in the Biological, Agri-Food and Forest Systems, Tuscia University, 01100 Viterbo, Italy; alessandro.rocci@unitus.it (A.R.); ilenia.baraccani@unitus.it (I.B.); eovidi@unitus.it (E.O.); 2 Research Institute on Terrestrial Ecosystems (IRET), National Research Council (CNR), 05010 Porano, Italy; chiara.anselmi@cnr.it (C.A.); dania.valli@cnr.it (D.V.); carlo.calfapietra@cnr.it (C.C.); 3 Department of Environmental Biology, Sapienza University of Rome, 00195 Rome, Italy; fabio.attorre@uniroma1.it

**Keywords:** agrobiodiversity, climate adaptation, particulate matter, urban agriculture, urban landscape design, *Vitis vinifera* L.

## Abstract

Urban vineyards are expanding worldwide, driven by cultural and environmental motivations and their potential contribution to sustainable food systems. This study assessed grapevine (*Vitis vinifera* L.) responses to urban atmospheric conditions and explored traits relevant to cultivar selection and particulate matter (PM) interception. Research was conducted during the 2025 growing season in a highly biodiverse urban vineyard comprising 170 cultivars at the Botanical Garden of Rome (Italy), a city where grapevine has been documented since antiquity. Digital phenotyping was used to screen 54 cultivars based on leaf micromorphological traits, followed by detailed assessment of five selected cultivars. Measurements also included leaf anatomical traits, chlorophyll content, spectral reflectance-derived indices related to vigor and water status, and PM interception by scanning electron microscopy. Cultivars differed in micromorphological traits and ecophysiological proxies, suggesting different responses to urban environmental conditions. Total leaf-surface PM accumulation after an approximately 20-day accumulation period following the last intense rainfall event (>10 mm h^−1^) ranged from 52,024 particles mm^−2^ in cv. Barbera to 77,462 particles mm^−2^ in cv. Maturano, values broadly comparable to those reported for some urban forest species. Deposition on the adaxial surface was more than twice that measured on the abaxial surface. These findings highlight the potential of digital phenotyping to integrate morphological and physiological traits, supporting plant material selection for urban viticulture, PM interception, and, more broadly, for urban landscape design.

## 1. Introduction

Urban landscapes are becoming increasingly productive. Urban food policies and the growing recognition of the multiple environmental, social, and cultural functions of agriculture in cities have increased the presence of agricultural biodiversity within urban areas [1,2]. Traditionally, agroecosystems mainly characterize peri-urban spaces, particularly in cities that have expanded by incorporating the surrounding countryside [3,4]. Today, many European metropolitan cities include large proportions of farmland within the urban functional area, where agroecosystems are recognized as components of urban green infrastructure, i.e., Nature-based Solutions (NbS) that provide multiple ecosystem services [2,5,6]. Given their key role in enhancing urban resilience and sustainability, in line with United Nations Sustainable Development Goal 11, the design and mapping of productive landscapes, regardless of their size, have become valuable tools for urban planning, governance, and public use [7].

Among the agroecosystems gaining importance in metropolitan areas are vineyards. In recent years, urban micro-vineyards have been established within cities, either in historical locations where vineyards once existed or in abandoned, degraded, or vacant urban spaces as multifunctional land uses for climate regulation, biodiversity enhancement, carbon storage, and wine production [8]. In fact, *Vitis vinifera* is used in urban green space design primarily as an ornamental species because of its high architectural and aesthetic value, resulting from its remarkable phenotypic plasticity, but interest in urban wine production has also grown substantially [9,10], reflecting its potential to support wine tourism and cultural wine routes, such as those promoted by the Council of Europe [11], as well as more sustainable ways of experiencing and exploring cities.

Urban vineyards represent an ideal system for identifying agricultural biodiversity capable of combining productive performance with the delivery of ecosystem services, thereby supporting evidence-based plant selection for productive NbS. Nonetheless, designing productive urban landscapes and supporting vine vegetative and reproductive development require knowledge of soil–plant–atmosphere relationships in the context of a stressful urban environment [12,13,14]. Despite the increasing spread of urban vineyards, little is known about the physiological performance of different grapevine cultivars under urban atmospheric conditions, particularly with regard to trade-offs between provisioning services (grape production) and regulating ecosystem services (climate regulation and air pollution mitigation). This knowledge gap limits the selection of suitable cultivars for urban productive landscapes.

Among the regulating ecosystem services potentially provided by urban vineyards, atmospheric pollutant mitigation remains one of the least investigated. Although plant–pollutant interactions have been extensively investigated for urban forest and forest species [15], they remain poorly understood in food-producing species within cities. The remarkable genetic diversity of agricultural species and cultivars, with grapevine accounting for more than one thousand known genotypes in Italy, suggests considerable potential for improving urban environmental quality through microclimate mitigation and atmospheric pollutant interception, although pollutant deposition may also affect crop quality and food safety, and should therefore be investigated.

Addressing the challenges posed by climate change requires interdisciplinary approaches and integrated technologies capable of making productive landscapes a structural component of urban ecosystems. In Rome (Italy), vineyards form distinctive cultural landscape patches within the city’s heterogeneous urban mosaic, bearing witness to a viticultural heritage that dates back to antiquity. Driven by the demand for grapes and wine since ancient times, vineyards were an integral component of the city’s landscape during the Roman Empire and remained widespread throughout the Middle Ages and the Renaissance [16,17]. During the twentieth century, however, many urban vineyards disappeared because of urban expansion and were progressively replaced by buildings, gardens, and other land uses, before being reintroduced through agrobiodiversity-based urban planning initiatives [1]. Supporting the design and conservation of urban productive vineyards not only helps preserve an iconic and culturally significant component of the city’s historical landscape, but also contributes to urban well-being through the multiple ecosystem services they provide. However, their implementation under current urban environmental conditions requires the identification and characterization of grapevine cultivars with traits potentially suited to changing environmental conditions.

The analysis of the local urban climate of Rome over the period 1989–2020 provided well-documented evidence of long-term trends in the city’s main climate variables, including a steady increase in mean annual temperature, higher mean temperatures across all seasons, an increased number of heat waves, and reduced rainfall, particularly during spring and summer, corresponding to a large part of the grapevine growing season [12]. These conditions may adversely affect grapevine vegetative growth and productivity. In addition, although the city’s air quality has recently shown an overall improvement, particularly since the COVID-19 pandemic, with PM10 and PM2.5 concentrations remaining below regulatory limits [18], further actions have been recommended to prevent future deterioration of air quality, also in the context of climate change mitigation. Climate change trends highlight the importance of selecting plant material potentially more resilient to changing climatic conditions for urban greening and urban agriculture projects.

To address these knowledge gaps, this study adopted an integrated, transdisciplinary methodology with two main objectives: (i) to evaluate grapevine responses to urban environmental conditions through the assessment of physiological proxies; and (ii) to assess the potential contribution of different cultivars to improving urban air quality through PM interception, using the city of Rome as a case study. We hypothesized that grapevine cultivars differ in their functional responses to urban atmospheric conditions, and that digital phenotyping, which provides the opportunity to quantify plant morphological and functional traits rapidly and non-destructively under field conditions, can support the identification of cultivars combining traits potentially relevant to provisioning and regulating ecosystem services.

## 2. Results

### 2.1. Climate and Particulate Matter Concentrations in the Research Areas

Overall, the climatic conditions of Rome, particularly in the urban area hosting the vineyard, showed potentially unfavorable features for grapevine cultivation, mainly related to the thermal regime and increased PM concentrations during prolonged periods without precipitation. The urban climate around the vineyard was characterized by the most common bioclimatic indices for viticulture (Table S1). The indices calculated for the 2025 growing season were compared with their long-term values for the reference period (2009–2024), and the main differences are highlighted in Table 1. The 2025 values showed a particularly high thermal availability and a higher occurrence of heat-stress days (+70% occurrence of days >30 °C). In contrast, the Cool night index (CI) before harvest was lower than the long-term average, indicating more favorable conditions for grapevine cultivation. Overall, these climatic conditions indicate potential heat stress, altered phenological development, and potential negative impacts on grapevine physiology and berry quality.

**Figure 1:**
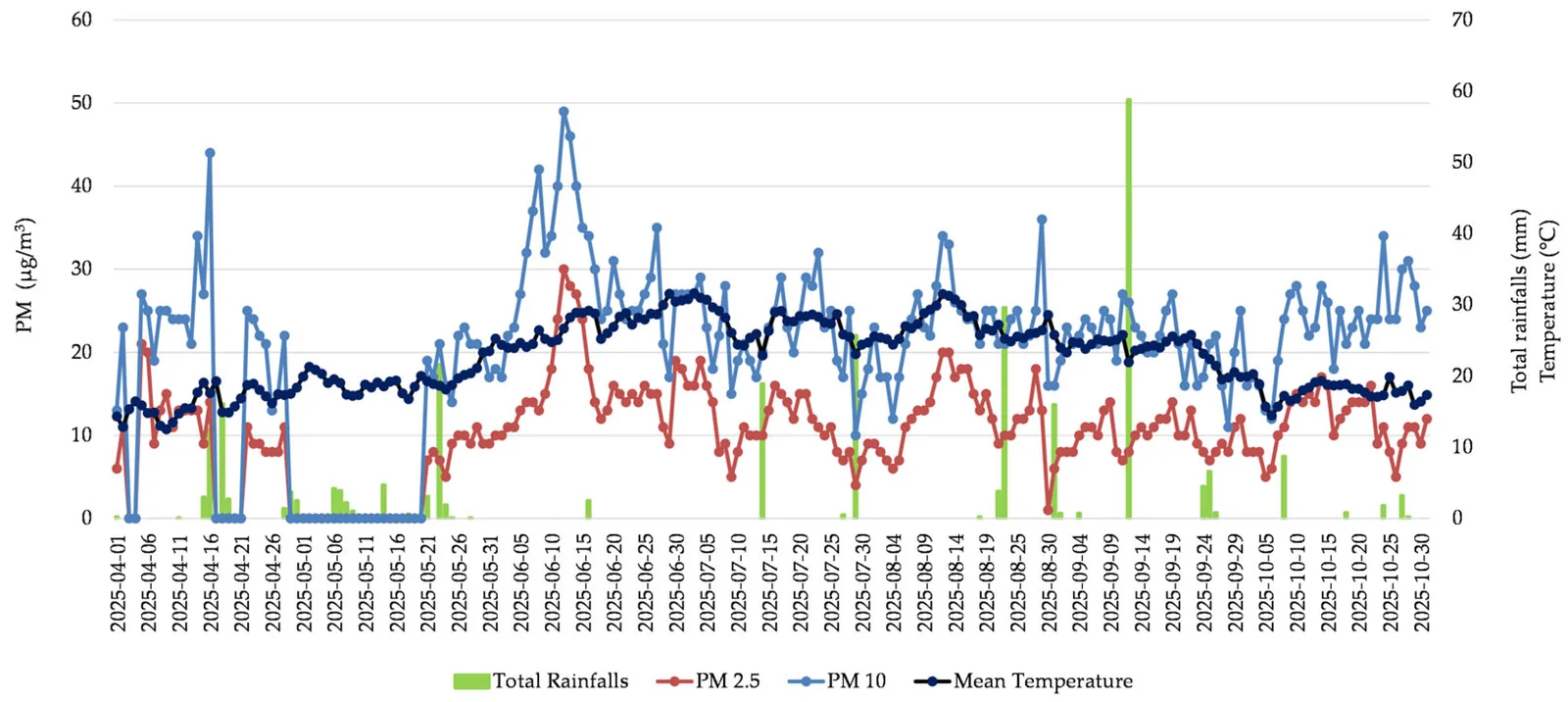
Daily particulate matter (PM2.5 and PM10) concentrations, mean temperature and total rainfall for the 2025 growing season (1 April–31 October) in the urban area of Rome.

Daily particulate matter (PM) concentrations, mean temperature and total rainfall (TR) in the urban area surrounding the vineyard during the 2025 grapevine growing season (conventionally 1 April–31 October) are shown in Figure 1.

Overall, during the growing season, the urban area showed high particulate levels: an average of 32.7 µg m^−3^ for PM10 and 21.5 µg m^−3^ for PM2.5. Total annual rainfall amounted to 581.3 mm.

PM10 peaks were particularly pronounced in the urban area during winter, reaching 201 µg m^−3^ on 6 December. PM2.5 followed the same pattern, reaching 160 µg m^−3^. Episodes above 50 µg m^−3^ for daily PM10 were frequent in the urban area proximal to the urban vineyard (54 days), highlighting persistent and intense particulate matter episodes outside the urban monitoring point during the observed period.

Data referring to the period immediately preceding leaf collection (Table S2) describe particulate matter accumulation during the interval following the last intense rainfall event recorded in the study area. During this interval (June–July 2025), pollutant concentrations were consistently high in the tested area.

### 2.2. Cultivar Variability in Ecophysiological Proxies Under Urban Conditions

Under urban conditions, all selected grapevine cultivars, including both native and international cultivars, maintained satisfactory chlorophyll levels throughout the growing season, while cultivar-specific differences in ecophysiological proxies emerged, providing potentially useful criteria for plant material selection in urban viticulture.

**Figure 2:**
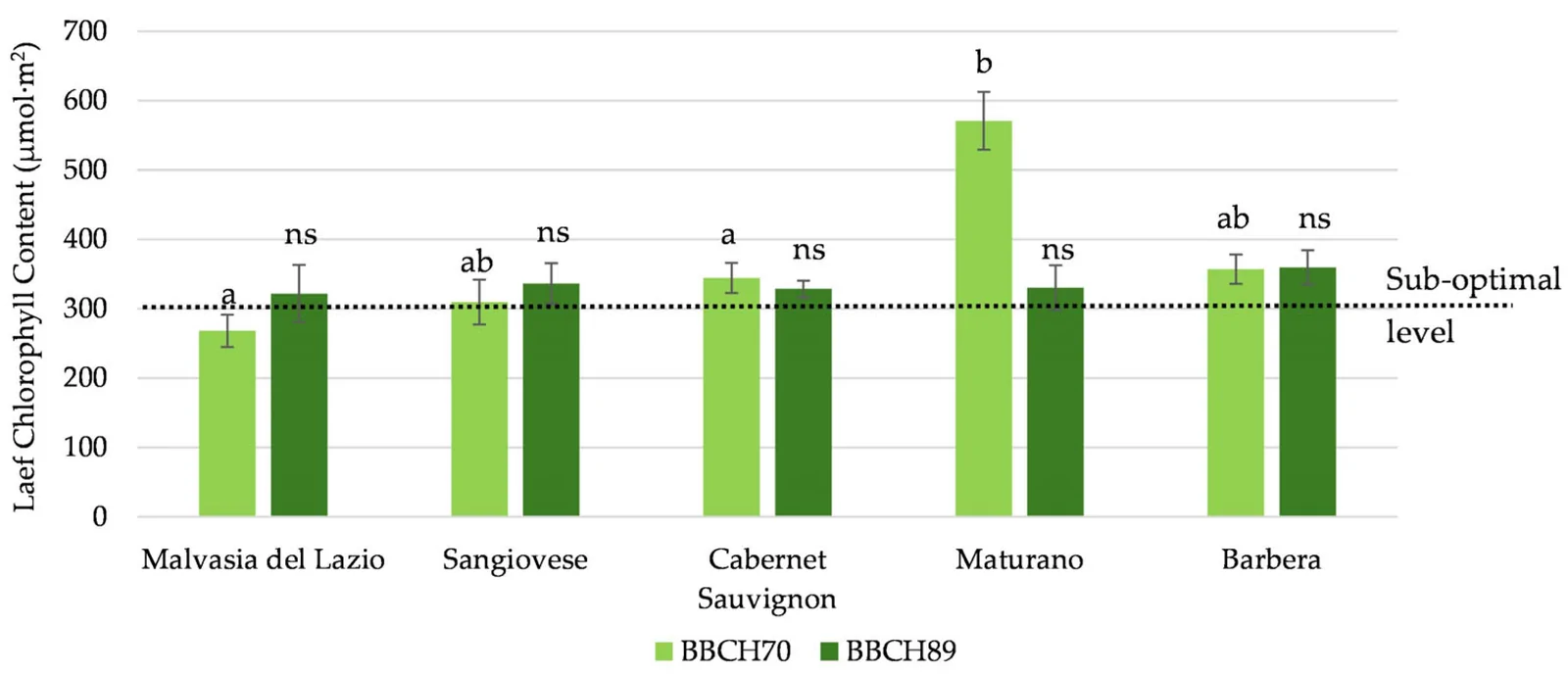
Leaf chlorophyll content (CHL) during the phenological stage BBCH70—fruit set (light green) and BBCH89—berries ripe for harvest (dark green) in the selected cultivars. Different letters indicate statistically significant differences according to the post hoc test; (ns) non-significant differences.

Grapevine had reached full leaf expansion, allowing a reliable comparison of leaf chlorophyll content among genotypes and sampling at two key phenological stages (fruit set and berry ripening). Differences were observed for leaf chlorophyll content, although, under urban conditions, it generally remained above the threshold reported in the literature as suboptimal for this species (300 µmol·m^2^) (Figure 2).

Chlorophyll content (CHL) at BBCH70 differed among the five grapevine cultivars (Kruskal–Wallis test: H(4) = 22.910, *p* < 0.001). Cv. Maturano showed the highest mean rank (27.500), whereas c v. Malvasia Lazio had the lowest (3.667). Dunn’s two-sided post-hoc comparisons, evaluated using the Bonferroni-corrected significance threshold (α = 0.005), indicated that cv. Maturano had higher CHL values than cvs. Malvasia del Lazio (*p* < 0.0001) and Cabernet Sauvignon (*p* = 0.004). No significant differences were detected for the remaining pairwise comparisons. On the other hand, the Kruskal–Wallis test revealed an overall difference in CHL at BBCH89 among cultivars (H(4) = 17.636, *p* = 0.001). Cultivars Barbera and Cabernet Sauvignon exhibited the highest mean ranks (23.833 and 22.333, respectively), whereas Sangiovese, Maturano, and Malvasia Lazio showed lower mean ranks (11.667, 12.786, and 13.071, respectively). Nevertheless, Dunn’s post-hoc test did not identify any significant pairwise differences after correction for multiple comparisons.

The same non-parametric Kruskal–Wallis test was used to assess differences among grapevine cultivars in the spectroradiometer-derived vegetation indices (Table 2). The Kruskal–Wallis test revealed cultivar differences in WI (*p* = 0.016) and NDII (*p* = 0.006), whereas PRI did not differ significantly among cultivars (*p* = 0.097). In particular, NDII was highest in Malvasia del Lazio (0.466) and lowest in Barbera (0.416). Dunn’s post-hoc test with Bonferroni correction confirmed a difference only between these two cultivars (*p* < 0.001), while all other pairwise comparisons were not significant. Overall, the findings highlighted differences among grapevine cultivars in water-related indices under the same urban abiotic conditions, suggesting cultivar-specific responses to environmental stress, particularly under heat and water stress conditions.

### 2.3. Leaf Morphological Traits

The study highlighted that grapevine leaf morphology encompasses multiple traits that can be assessed for their potential role in regulating plant–atmosphere interactions. The tested cultivars differed for some leaf morphological and stomatal traits (Table 3).

Significant differences were observed among cultivars for leaf area (Kruskal–Wallis, *p* = 0.040) and perimeter (Kruskal–Wallis, *p* = 0.011), which depend on genetic factors but can also be affected by environmental conditions. In particular, after Bonferroni correction, Dunn’s post-hoc test showed that cv. Cabernet Sauvignon had the smallest values, while cv. Maturano showed the highest values for both parameters. The aspect ratio was generally close to 1.0 for all cultivars, indicating relatively balanced leaf proportions, with no significant differences among genotypes (Kruskal–Wallis, *p* = 0.199).

Regarding stomatal density, no significant differences were detected among cultivars (Kruskal–Wallis, *p* = 0.059), indicating broadly comparable values across the analyzed cultivars. Stomatal size-related parameters were also investigated, and significant differences were observed among cultivars in the semi-major axis (Kruskal–Wallis, *p* < 0.001), with cv. Maturano showing the smallest stomatal dimensions. Significant differences were found among cultivars in linear distance between stomatal apertures (Kruskal–Wallis, *p* = 0.011).

Overall, the results highlight substantial cultivar-dependent variability. Cultivars such as Maturano combine large leaf areas with relevant morphological traits that may support PM interception, while cv. Malvasia del Lazio stands out for its particularly high stomatal density. These differences should be considered when evaluating the role of grapevine cultivars in particulate matter capture and environmental biomonitoring.

Another leaf morphological trait that can contribute to PM retention is leaf roughness (Table 4).

The selected cultivars in the urban vineyard of Rome showed variation in SEM-derived image-texture descriptors (Table 4), including differences between the abaxial and adaxial surfaces. These descriptors reflect comparative variations in grayscale intensity and should not be interpreted as direct measurements of physical surface roughness, height, or depth.

On the abaxial leaf surface, cv. Malvasia del Lazio showed the highest values of Rq and Ra—286.92 and 220.55, respectively—indicating the highest values of these grayscale-based texture descriptors among the cultivars. Cultivars Maturano and Barbera had lower Ra, indicating comparatively lower grayscale-intensity variation on their abaxial surfaces. However, cv. Barbera showed the highest Rp and Rt values, with Rp = 1316.95 and Rt = 2016.65, indicating greater variation in the corresponding image–based texture descriptor. The Rv values did not differ among cultivars on the abaxial side, as all cultivars shared the same statistical letter.

On the adaxial leaf surface, cv. Barbera again showed the highest image-texture descriptors. These values indicate comparatively greater grayscale-intensity heterogeneity in the SEM images of cv. Barbera, rather than greater physical surface roughness or topographic relief. In contrast, cv. Maturano had the lowest Rq, Ra, Rp and Rt values, suggesting a lower variation in the corresponding image-texture descriptions. The higher Rv value observed in cv. Barbera represents a higher value of this specific image-texture descriptor.

### 2.4. Particulate Interception

The results showed substantial PM interception by leaves of all tested grapevine cultivars, with greater accumulation on the adaxial leaf surface.

**Figure 3:**
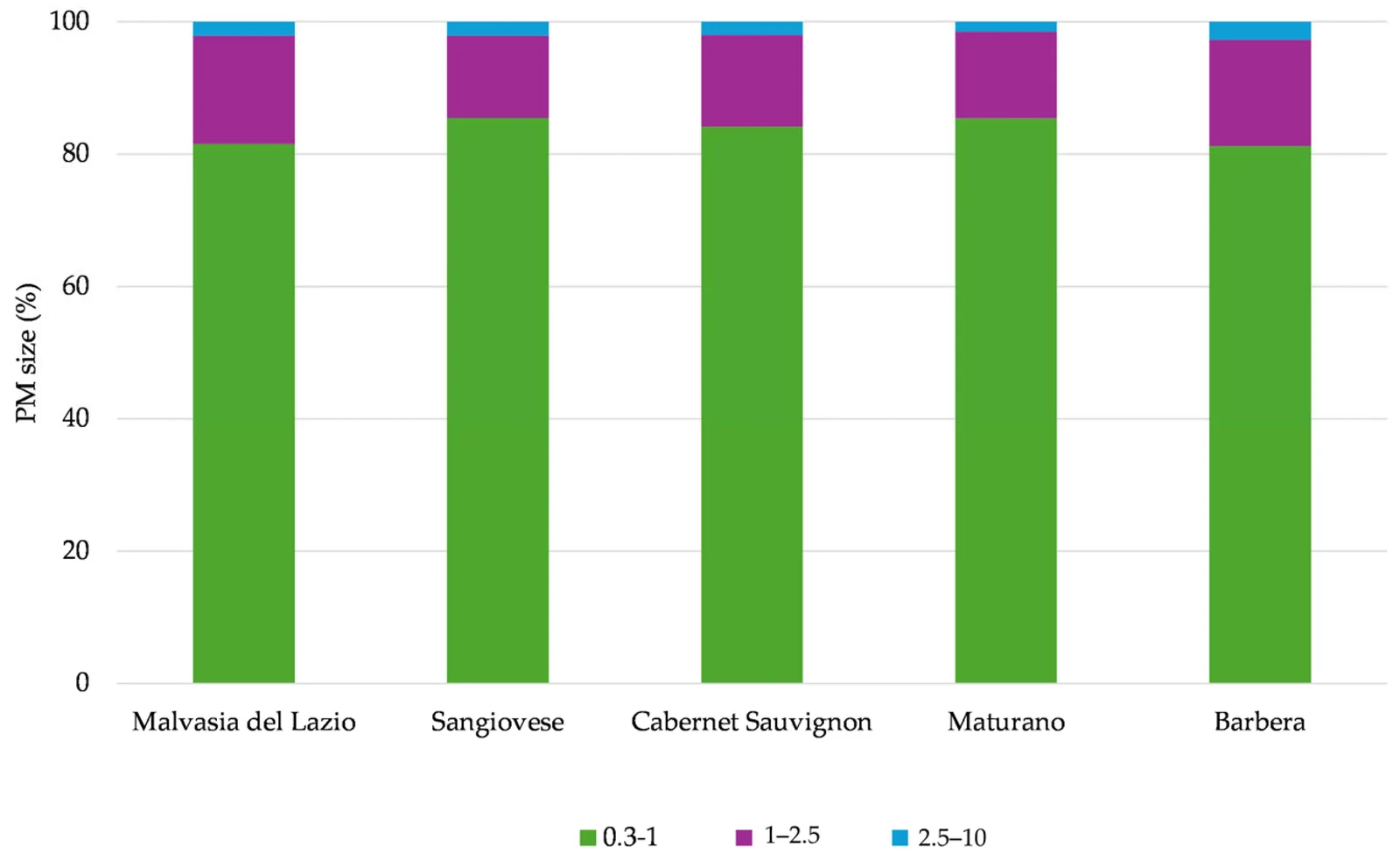
Percentages of particles accumulated on grapevine leaves in the different PM classes in the cultivars Malvasia del Lazio, Sangiovese, Cabernet Sauvignon, Maturano and Barbera, cultivated in the urban vineyard of the Botanical Garden of Rome.

PM accumulation data showed a similar particle-size distribution across all analysed cultivars, with the ultrafine fraction (0.3–1 µm) accounting for at least 80% of the accumulated particulate matter, and the coarse fraction (2.5–10 µm) representing less than 5% of the total (Figure 3).

**Figure 4:**
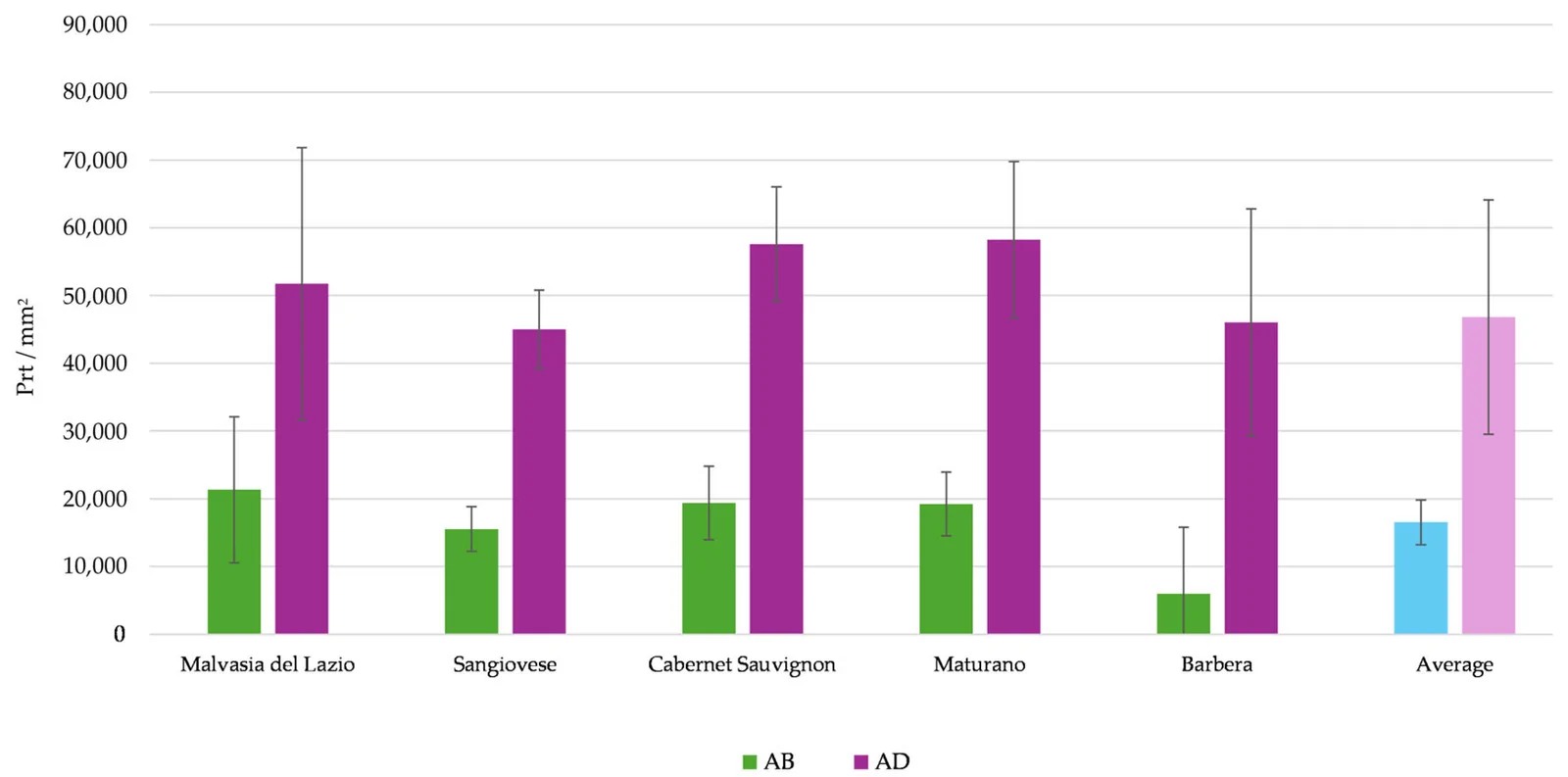
Particles (number) per mm^2^ accumulated on the adaxial (purple) and abaxial (green) surface of grapevine leaves of tested cultivars: Malvasia del Lazio, Sangiovese, Cabernet Sauvignon, Maturano, Barbera and average values of particles per mm^2^ accumulated on the adaxial (pink) and abaxial (light blue) surface of *Vitis vinifera* L. cultivated in the urban vineyard of the Botanical Garden of Rome.

Differences in accumulation between the adaxial and abaxial leaf surfaces were evident in all tested cultivars under urban conditions (Figure 4), with higher accumulation on the adaxial side; the abaxial surface accumulated less than half as much as the adaxial surface. However, there were no significant differences among cultivars in retention on either leaf surface.

The exploratory Spearman correlation matrix calculated from the five cultivar-level means is reported in Table 5; it contains pairwise correlation coefficients for some selected variables among morphological, physiological and particulate-matter data. Positive correlations are shown in blue and negative correlations in red; darker colours indicate stronger relationships. The correlation matrix revealed several strong associations. Leaf area (LA) showed a moderate positive association with leaf perimeter (LP) and a strong negative association with stomatal semi-major axis. Leaf perimeter was also positively associated with stomatal density and negatively associated with stomatal semi-major axis, indicating that higher LA and LP values tended to be associated with lower S-mA values, while LP and SD generally varied in the same direction. A strong positive correlation was observed between NDII and AB-Ra. Total PM accumulation on the two leaf surfaces (PM TOT AB and PM TOT AD) was also strongly and positively correlated, indicating similar relative variation between the two leaf surfaces. Stomatal density showed positive associations with total PM accumulation on both the abaxial and adaxial surfaces, although these associations were comparatively weak. Among the surface-related variables, AB-Rv was strongly and negatively correlated with AB-Rp, whereas AB-Rp was strongly and positively correlated with AD-Ra. The strongest association observed in the matrix was between AD-Rv and AD-Rp, which displayed an almost perfect negative correlation. Several additional associations were observed, including SD with NDII, AB-Ra with AB-Rp, PM TOT AB with AD-Rv, and AD-Ra with AD-Rv, and PM TOT AB with NDII.

The observed coefficients are therefore reported as exploratory patterns rather than confirmatory evidence of trait–function relationships.

## 3. Discussion

There is renewed interest in NbS for European cities following the recent adoption of the Nature Restoration Law [19], which establishes restoration targets for degraded ecosystems, including urban ecosystems, and requires restoration of at least 20% of the EU’s terrestrial areas by 2030, and all ecosystems in need of restoration by 2050. Cities can be considered complex biospheres where infrastructure, humans, animals, and plants coexist and interact with environmental matrices. Appropriate management of these interactions can drive the transition towards biocities [20]. In this context, productive landscapes, namely the use of food-producing species to provide multiple ecosystem services, including cultural services, both as physical spaces and as ecological processes, are gaining increasing attention as a means of reintroducing or strengthening the presence of nature into cities. This study provides exploratory evidence on cultivar-specific responses to the urban environment and on the potential for a functional classification of plant material for use in NbS based on physiological proxies and morphological traits.

### 3.1. Rome’s Urban Vineyards

The grapevine (*Vitis vinifera* L.), one of the earliest fruit crops domesticated in the Mediterranean [21], has been an integral component of Rome’s landscape since antiquity. Historical and archaeological evidence documents its widespread presence throughout the city, where vineyards have long contributed to both agricultural production and the cultural identity of the urban landscape. Today, vineyards occupy both historical locations and newly established urban sites, but their role has evolved from being predominantly productive to increasingly multifunctional, providing environmental, cultural, and social benefits alongside grape and wine production.

The recent expansion of urban vineyards in Rome reflects multiple complementary objectives. While vineyards newly planted within archaeological and historical sites primarily aim to preserve the cultural identity and *genius loci* of the archaeological landscapes, more recent urban vineyard projects are increasingly intended to contribute to urban sustainability and regeneration. By transforming underused and degraded spaces into productive green infrastructure, urban vineyards can enhance ecological connectivity, support biodiversity, and deliver multiple ecosystem services. In this context, *Vitis vinifera* has emerged as a particularly versatile species for urban ecological design, combining adaptability to different environmental conditions with cultural significance and the capacity to support NbS across a wide range of urban settings [1].

The vineyard of the Botanical Garden of Sapienza University of Rome is one of several vineyard sites embedded within the urban fabric, either as remnants of the ancient *ager Romanus* or as recently established vineyards, such as those at the Colosseum Archaeological Park (Barberini vineyard) [22] and within the archaeological area of the Baths of Caracalla (San Sisto vineyard). Together, these vineyards contribute to the distinctive identity of Rome, the largest agricultural municipality in Europe, with more than 61,000 ha of agricultural land. The vineyard investigated in this study, however, is unique because of its exceptional genetic diversity, comprising 170 *Vitis vinifera* accessions, and because it has become a living laboratory for investigating the responses of grapevine agrobiodiversity to urban environments [23,24,25].

Producing high-quality grapes in urban environments requires the integration of site-specific knowledge, appropriate cultivar selection, and ecophysiological and agronomic expertise. The grapevine germplasm collection maintained at the Botanical Garden has provided a unique opportunity to evaluate cultivar behavior under multiple abiotic stressors typical of urban environments, including elevated temperatures, drought, and atmospheric particulate matter. Many other grapevine responses to urban environments remain underinvestigated, although understanding them is necessary to ensure adequate vegetative and reproductive growth. The present findings highlight that scientific knowledge and technological innovation are essential for identifying cultivar-specific traits potentially relevant to plant material selection for urban viticulture under current and future climate scenarios. This unique combination of historical continuity, exceptional genetic diversity, and urban environmental exposure makes this urban vineyard an ideal living laboratory for investigating grapevine responses to multiple urban stressors and for developing science-based strategies for productive urban green infrastructure.

### 3.2. Vine-Urban Atmosphere Interactions: Climate and Air Particulate

Climate change is increasingly threatening not only human well-being but also the biodiversity that supports ecosystem functions contributing to mitigation of the urban microclimate. Consequently, climatic variables should be evaluated in relation to their potential effects on primary plant physiological processes and, consequently, on vegetative growth and reproductive development. Grapevine is highly sensitive to climatic conditions, particularly with respect to phenology, grape ripening and composition. For this reason, it is widely recognized as a bioindicator of climate change, and several bioclimatic indices based on temperature and precipitation have been developed to assess site suitability for grape and wine production [26]. The climatic analysis of the study area revealed warmer conditions in 2025 compared with the mean of the previous 15-year period, accompanied by a high frequency of extreme heat events and low rainfall, resulting in multiple potential summer stressors. These conditions may affect grapevine growth and increase the risk of yield and quality losses unless appropriate climate adaptation strategies are adopted [27,28].

Moreover, urban environments expose grapevines to additional stressors, particularly atmospheric particulate matter (PM), which may further affect plant physiological performance. To date, these aspects have been investigated primarily in urban forests and ornamental trees [29,30,31] and, to a lesser extent, in vertical greening systems, which can also provide multiple ecosystem services, including thermal insulation, urban cooling, noise attenuation, biodiversity conservation, and improvements in local air quality [32,33,34]. Urban trees have been extensively studied with respect to PM deposition and pollutant removal in relation to canopy structure and leaf functional traits [15,35,36]. Recent evidence also indicates that climate extremes and declining climatic suitability may threaten the persistence of urban forests and reduce their capacity to provide ecosystem services, although vulnerability varies considerably among species and locations [37]. In addition, studies on living walls have demonstrated that plant responses to urban environmental conditions are strongly species-dependent and that combinations of functional traits—including leaf size, surface roughness, trichome density, epicuticular waxes, leaf rigidity, and canopy density—can substantially influence PM capture and retention. Reviews of vertical greening systems similarly emphasize that species selection should be based on functional traits rather than on ornamental value alone [38]. In the study by Weerakkody et al. [39], PM capture differed significantly among seventeen living-wall species. Smaller-leaved species with waxy or structurally complex surfaces generally exhibited greater particle retention, although overall performance also depended on leaf area index and canopy characteristics.

Deposition efficiency is also affected by local PM concentrations, source type, wind conditions, precipitation, exposure duration, and canopy position. Steinparzer et al. [40] demonstrated that PM accumulation within the canopy is jointly influenced by leaf habit, urbanization, and pollution level. Consequently, differences among studies should not be attributed exclusively to plant traits. However, in the present study, all grapevine cultivars were grown at the same site and sampled under comparable environmental conditions, which reduced variation associated with pollution exposure and allowed cultivar-related differences to be examined under the same urban environmental conditions.

Particle-number densities measured on grapevine leaves fall within the broad range reported for urban woody vegetation [41], including *Fraxinus ornus*, *Celtis australis*, *Tilia cordata*, and *Morus alba*, and are similar to those reported for deciduous broadleaf trees such as *Populus alba*, *Ginkgo biloba, Q. robur* and *Q. petraea*, and *Tilia cordata* [42], which ranged from 202 to 10,981 particles mm^−2^ during the June sampling campaign. Fine particles were substantially more abundant than coarse particles, and the adaxial leaf surface accumulated approximately 2.6 times as many particles as the abaxial surface. This is consistent with the pattern observed in the investigated grapevine cultivars, in which the abaxial surface retained less than half the number of particles detected on the adaxial surface. The greater adaxial load may be associated with its direct exposure to gravitational settling and atmospheric particle flux. Conversely, the position of the abaxial surface may partially shield it from direct deposition. In a Mediterranean study conducted in central Genoa [43], marked interspecific differences in particle-number density were observed, with *Trachelospermum jasminoides* and *Hedera helix* displaying greater retention than *Cistus ‘Jessamy Beauty’* and *Phlomis fruticosa*, with values comparable to those found for *Vitis vinifera* L.

Overall, these comparisons indicate that the particle densities observed on grapevine leaves are within the range reported for urban vegetation. Nevertheless, absolute values cannot be interpreted exclusively as species-specific retention capacities because particle loading is also affected by source intensity, exposure duration, rainfall, wind, and sampling season.

Only a few studies have examined the relationship between agrobiodiversity and particulate matter. In Mediterranean cities, for example, bitter orange (*Citrus aurantium* L.) is widely planted along streets, in pedestrian areas, and in public spaces. Its evergreen canopy has also been used as a passive biomonitor of traffic-related and other airborne contaminants [44,45]. Such examples suggest that perennial crop species may tolerate prolonged exposure to urban conditions while providing persistent foliar surfaces capable of intercepting airborne pollutants. Nevertheless, their ecosystem-service potential cannot be inferred solely from their survival or traditional ornamental use.

Species and cultivar selection should therefore be based on integrated assessments of tolerance to urban stress, ecophysiological responses, canopy development, leaf functional traits, pollutant interception and retention, management requirements, and, in the case of edible species, productive performance, product quality, and food safety. This is particularly important because PM capture varies markedly among species and depends not only on individual leaf traits but also on canopy architecture, leaf exposure and orientation, local environmental conditions, and the potential for particle resuspension or wash-off [38,39]. Within this framework, woody perennial crops such as grapevine may represent a valuable but still underexplored option for productive urban greening. Grapevine is especially relevant because of its high intraspecific diversity, which provides considerable variation in morphology, phenology, water-use strategies, and physiological responses to environmental stress. The cultivation of *Vitis vinifera* L. is based on an extraordinary genetic diversity, with more than 600 cultivars registered for cultivation and transformation in Italy [46], compared with other fruit species that have a much more restricted cultivar assortment. Investigating its ecophysiological resilience and pollutant-interception capacity at the cultivar level is therefore essential for identifying genotypes that are suited to urban environments and potentially capable of combining food production with regulating ecosystem services, including PM retention. However, even within urban forestry research, measurements of deposited PM are not always accompanied by detailed assessments of plant physiological status, long-term tolerance, or the consequences of chronic pollutant exposure. This knowledge gap is even more pronounced for perennial food crops, for which productive performance, product quality, and food safety must be evaluated alongside regulating ecosystem services.

In particular, the ecophysiological responses of urban vineyards to the combined effects of climatic stress and atmospheric particulate matter remain poorly understood, despite the growing importance of grapevines as multifunctional components of urban green infrastructure.

### 3.3. Digital Phenotyping

Digital phenotyping is a powerful means of acquiring the knowledge necessary for a systemic evaluation of multiple plant traits and responses under urban environmental conditions. In this study, it proved to be a useful, non-destructive approach for evaluating grapevine performance under urban conditions. The integration of chlorophyll measurements, hyperspectral reflectance, water-related vegetation indices, stomatal traits, and digital analysis of leaf morphology enabled the discrimination of cultivars growing under identical environmental conditions, supporting the use of these techniques as rapid screening tools for large grapevine collections. Such an approach may shift cultivar selection from a predominantly production-oriented perspective towards a multifunctional framework that simultaneously considers physiological responses, environmental performance, productivity, and possible ecosystem-service provision.

Very little is known about the ecophysiological performance of woody crop species under prolonged unfavorable urban atmospheric conditions or about their capacity to simultaneously support food production and provide regulating ecosystem services. Digital phenotyping provides a useful, highly sophisticated tool for rapid, non-destructive assessment of ecophysiological responses and morphological traits [47]. In particular, it enables precise analysis of plant morphological traits, such as leaf morphology, a key factor in vine–atmosphere interactions and in the provision of regulatory ecosystem services (e.g., microclimate regulation, carbon storage, and pollutant interception). Furthermore, integrating digital morphological and physiological data provides comprehensive insights into plant responses under changing climatic conditions.

The present study addresses this knowledge gap by providing an integrated assessment of grapevine-atmosphere interactions in an urban vineyard through the combined use of digital leaf phenotyping, ecophysiological proxy measurements, and SEM characterization of deposited PM. During the 2025 growing season, grapevines were exposed to average atmospheric PM10 and PM2.5 concentrations of approximately 32.7 and 21.5 µg m^−3^, respectively. Under these common environmental conditions, cultivars showed differences in water-related spectral indices, leaf morphology, stomatal traits, surface image-based texture descriptors, and PM accumulation patterns, whereas leaf chlorophyll content remained generally stable. The maintenance of satisfactory chlorophyll content suggests that photosynthetic function was not markedly affected, at least in terms of pigment status, despite the combined effects of particulate pollution, elevated temperatures, and potential water limitation. Furthermore, water-related spectral indices revealed cultivar-specific differences in water status. Cv. Sangiovese showed the highest Water Index values, whereas cv. Cabernet Sauvignon exhibited the lowest values. Similarly, NDII distinguished cultivars, with the highest values in cv. Malvasia del Lazio and the lowest in cv. Barbera, while PRI did not differ significantly, indicating that structural and water-related responses were more pronounced than short-term photochemical adjustments.

These findings are consistent with previous evidence showing that PM deposition may alter epicuticular waxes, partially obstruct stomata, modify leaf optical properties, and interfere with photosynthesis and plant water relations. The absence of chlorophyll decline should therefore not be interpreted as the absence of stress, since changes in stomatal conductance, chlorophyll fluorescence, oxidative metabolism, or carbon assimilation may occur before visible pigment loss [48]. The PM density values observed for each cultivar suggest the potential of grapevine to intercept and retain atmospheric PM, with values comparable to those reported for trees or evergreen shrubs [40,41,42,43]. Deposition on the adaxial leaf surface was more than twice that measured on the abaxial surface, suggesting that particle interception depends on leaf exposure, orientation (e.g., in different training systems), and surface characteristics [15,39].

Moreover, the observed cultivar differences cannot be attributed solely to PM exposure but rather reflect integrated responses to multiple urban stressors, including elevated temperatures, water limitation, atmospheric pollution, and local environmental conditions. All cultivars intercepted atmospheric particles across the investigated size classes. Although PM 0.3–1 accounted for more than 80% of the total particle number, the larger PM 2.5–10 fraction contributed disproportionately to the estimated particle mass. However, particle deposition should be interpreted as evidence of interception and temporary retention rather than permanent removal, since deposited particles may subsequently be resuspended by wind or removed by rainfall.

The exploratory correlation analysis provided initial information on the possible relationships between cultivar-level leaf traits and PM interception. Leaf-surface complexity is generally considered capable of promoting particle interception by providing depressions, irregularities and protected microsites in which deposited particles may be retained. However, the effects of individual surface traits vary among species and environmental conditions, and PM retention generally results from the combined influence of surface structure, epicuticular waxes, trichomes, stomata, leaf orientation and atmospheric exposure [39].

Overall, the observed differences among cultivars suggest differences in adaptability potential rather than definitive adaptation to urban environments. In this context, acclimation refers to phenotypic and physiological adjustments occurring within the lifetime of individual plants, whereas demonstrating genetic adaptation would require evidence of heritable responses across generations. Consequently, long-term assessments conducted over multiple growing seasons and urban environments will be necessary to confirm cultivar suitability for sustainable urban viticulture.

These results indicate that grapevine responses to urban conditions are strongly cultivar-dependent and highlight the potential of functional phenotyping for selecting cultivars potentially suited to urban viticulture.

### 3.4. Implications and Future Perspectives

Urban vineyards have acquired multiple functions beyond grape production. In addition to representing culturally valuable urban landscapes, they offer opportunities to develop plant-based strategies for climate adaptation and to serve as open-air living laboratories for urban sustainability research. Food production within the urban ecosystem is part of these strategies. The increasing impacts of climate change, particularly evident in the Mediterranean Basin, where the frequency and intensity of extreme climatic events are rising [49,50], highlight the urgent need to bridge current knowledge gaps regarding the adaptation of food crops to urban environments and their potential contribution to improving urban environmental quality. The study provided exploratory indications of the potential of *Vitis vinifera* L. to contribute to ecosystem service provision, although it addressed only a few of the services that agrobiodiversity may provide in urban environments, i.e., PM interception and, indirectly, photosynthetic performance, which underpins carbon assimilation and storage. The restricted number of biological replicates and cultivars evaluated may also represent a limitation of the study, since the five individual cultivars cannot be considered representative of their entire morphological groups, given the much broader phenotypic variability characterizing grapevine cultivars. Nevertheless, the results may provide a basis for further research to address remaining knowledge gaps, for instance in terms of carbon storage, urban soil fertility, and fruit quality. These aspects are currently under investigation.

Finally, the planning and design of productive urban landscapes cannot rely solely on traditional horticultural or aesthetic criteria. Scientific knowledge and technological innovation are essential for supporting evidence-based urban agriculture and ecological design. In this context, selecting the right genotype for the right place becomes a key design principle. Beyond survival under stressful conditions or ornamental value, cultivar selection should increasingly consider functional traits associated with physiological resilience and multiple ecosystem-service provision. Digital phenotyping, by integrating ecophysiological, morphological, and functional traits, offers a powerful approach for developing new classification frameworks that support the selection of cultivars potentially suited to sustainable and resilient urban landscapes.

## 4. Materials and Methods

### 4.1. Study Sites and Urban Climate Assessment

**Figure 5:**
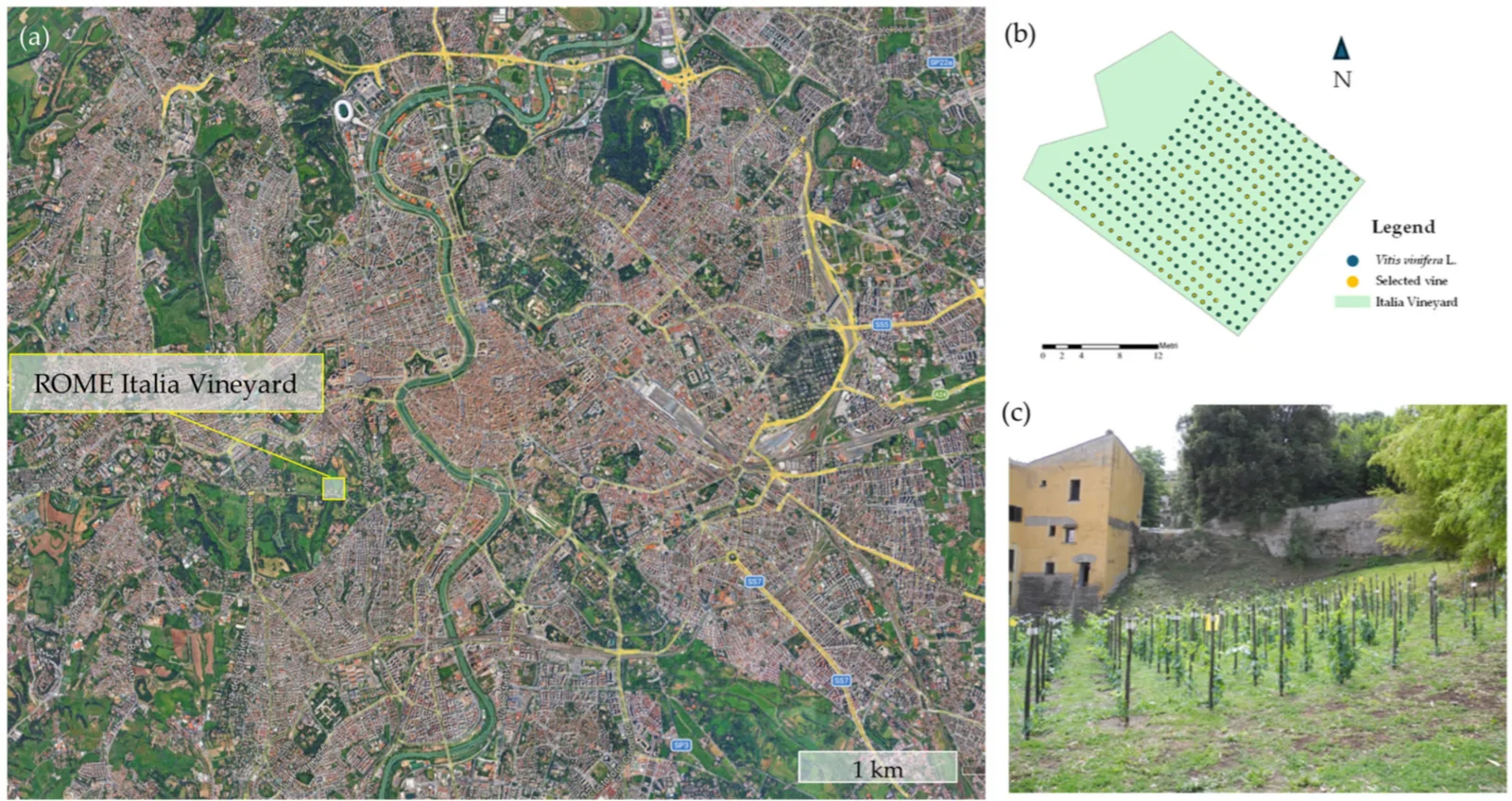
Location of the tested vineyard in the metropolitan area of Rome, Central Italy (**a**). The urban site is represented by “Italia Vineyard” within the Botanical Garden of Rome, where 170 cultivars of *Vitis vinifera* L. (**b**) were cultivated under organic management and “albarello” training system (**c**).

The study was carried out during the 2025 growing season in the urban vineyard located within the Botanical Garden of Rome (Central Italy) (WGS84 Lat:41.889932; Long:12.465023), named “Italia Vineyard”, a core collection of Italian grapevine cultivars (Figure 5). This 600 m^2^ micro-vineyard hosts 155 Italian native grapevine cultivars representative of the 20 Italian regions, together with 15 international varieties, all represented by two vines. This site was selected because of its urban location, which allows the assessment of grapevine ecophysiological responses under abiotic stressors, while its high genetic variability enables comparison of particulate matter (PM) retention among autochthonous and nationally relevant cultivars.

The climatic conditions of the study site were characterized through the calculation of the main bioclimatic indices used in viticulture (Table S1), according to Biasi et al. [26], and the differences recorded in 2025 relative to the long-term average (years 2009–2024) were considered in relation to their potential suitability for grapevine cultivation and grape quality but were not intended to explain or to establish correlations with the physiological responses of the different cultivars observed during the 2025 growing season. Meteorological data were provided by the regional agrometeorological network stations closest to the urban vineyard: the ‘Lanciani’ weather station (WGS84 Lon: 12.52352; Lat: 41.92047). The recorded variables included air temperature, relative humidity, and rainfall. Air quality was assessed using data from the nearest air-quality monitoring stations of the Regional Environmental Protection Agency of Lazio (ARPA Lazio), located approximately 800 m (WGS84 Lon: 12.475387; Lat: 41.893971) from the vineyard. Airborne PM data for PM (2.5–10 μm) and PM > 10 (diameter >10 μm) size classes were used to characterize the environmental pollution pressure during the growing season and to identify the summer peak in PM exposure [51].

### 4.2. Digital Phenotyping of Leaves, Plant Material, Cultivar Selection, and Sampling

The digital phenotyping approach adopted in this study integrated image-based morphological traits, chlorophyll measurements, spectral reflectance-derived indices, and PM retention. Digital phenotyping followed a multiscale approach, starting with a broad assessment of leaf morphological and micromorphological traits in the initial set of 54 cultivars (first-level digital phenotyping), followed by a more detailed characterization of the five selected cultivars, including chlorophyll measurements and spectral reflectance-derived indices (second-level digital phenotyping). Final cultivar selection followed a functional criterion based on leaf surface traits, which can influence plant–atmosphere interactions and the plant’s response to environmental conditions, while also potentially contributing to the interception of atmospheric PM [52].

**Figure 6:**
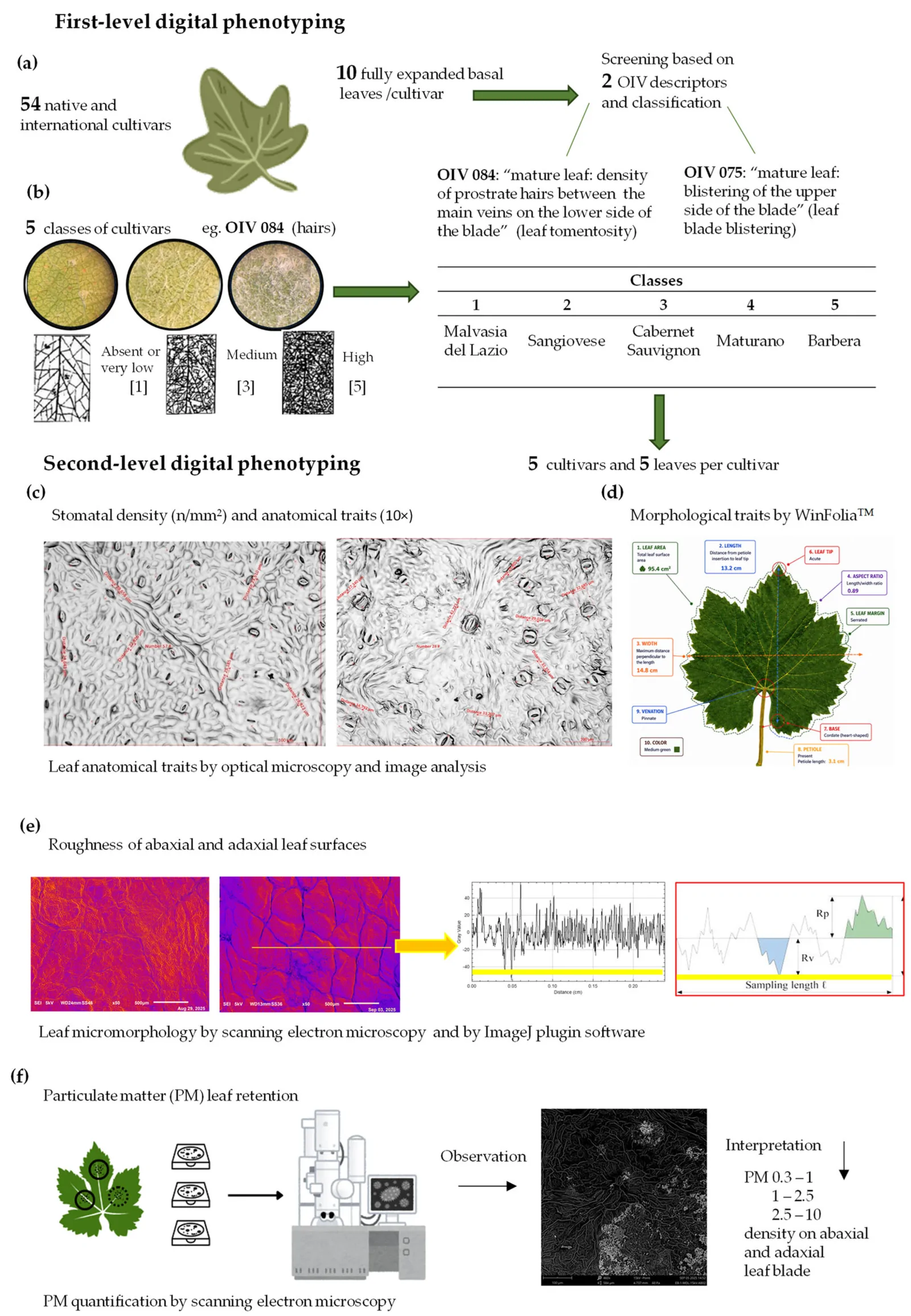
Schematic representation of the leaf digital phenotyping approach used on 54 cultivars of the urban vineyard of Rome. (**a**) Ten basal leaves per cultivar were analyzed for two ampelographic descriptors of the International Organisation of Vine and Wine (OIV), i.e., OIV 084: “mature leaf: density of prostrate hairs between the main veins on the lower side of the blade” (leaf tomentosity) and OIV 075: “mature leaf: blistering of the upper side of the blade” (leaf blade blistering), (**b**) used to classify all grapevine genotypes into five groups and then select 5 cultivars of interest; further digital phenotyping included stomatal traits (**c**), detailed leaf morphological traits (**d**), leaf blade texture descriptors (**e**), and leaf PM retention (**f**).

The initial screening included 54 cultivars, selected from the 170 available in the collection. These cultivars were chosen to represent Italian autochthonous grapevine diversity across different geographical areas (Northern, Central and Islands, and Southern Italy) and berry colour (black and white), together with some international cultivars. These cultivars were then classified into five classes according to two ampelographic micromorphological descriptors of the International Organisation of Vine and Wine [53]: OIV 084: “mature leaf: density of prostrate hairs between the main veins on the lower side of the blade”, here referred to as leaf tomentosity; and OIV 075: “mature leaf: blistering of the upper side of the blade”, here referred to as leaf blade blistering (Figure 6a). These two descriptors, which potentially affect PM deposition and retention, were used to classify the genotypes into five morphological groups based on increasing levels of leaf tomentosity and blade blistering (Figure 6b). Within each group, one cultivar was selected for detailed digital phenotyping. The final selection prioritized cultivars native to the Lazio region, considering the location of the study in Rome, together with the widely cultivated international cv. Cabernet Sauvignon, resulting in the final set of five cultivars. The five selected cultivars were: cv. Malvasia del Lazio (MDL), cv. Sangiovese (SAN), cv. Cabernet Sauvignon (CAB), cv. Maturano (MAT), and cv. Barbera (BAR). This selection was intended to increase the practical relevance of the study for the development of urban vineyard projects based on locally adapted plant material, rather than to generalize the physiological behavior of each selected cultivar to the entire morphological group.

Two vines per cultivar were analysed in the study. The two vines were of the same age and grown under uniform management conditions. Since the vineyard is a germplasm collection in which each cultivar is represented by only two individuals, no additional vines of the same cultivar were available as experimental units and for sampling.

For each cultivar, ten fully expanded basal leaves were randomly collected from comparable canopy positions, at a height of 70–80 cm above the ground. The leaves were used as sampling units for the morphological, physiological, and PM assessments, while repeated measurements performed on the same leaf were averaged. Leaves were sampled (8 July 2025) at least 15 days after the last intense rainfall event, defined as rainfall intensity greater than 10 mm h^−1^, to allow PM accumulation on the leaf surface, and sampling was performed during the summer peak of airborne particulate matter, as indicated by regional air-quality ARPA monitoring stations. Five leaves were used to quantify PM accumulation and stored in clean paper bags, avoiding contact with the container surfaces; the bags were then sealed. The remaining five leaves were processed fresh for morphological analysis.

### 4.3. Leaf Micromorphological Trait Assessment

Leaf morphological traits were quantified using digital image analysis. Leaf area, leaf shape, margin characteristics, and aspect ratio, calculated as the ratio of leaf blade length to width, were measured using WinFOLIA™ software version 2022 (Regent Instruments Inc., Quebec, Canada) (Figure 6d). On the same leaves, stomata density was assessed from imprints obtained by applying nail polish to the abaxial leaf surface, after trichomes had been removed using clear adhesive tape, to obtain clearer microscopic images of the stomata. The dry nail polish film was then peeled off using clear tape (Crystal Clear Office Tapes, supplied by Tape Providers, Hialeah, FL, USA) and placed on a microscope slide. Three microscopic slides were prepared from each leaf avoiding the major veins. Stomata were then observed by an Axioskop 2 plus conventional microscope (Carl Zeiss, Oberkochen, Germany) equipped with the Axiocam 503 color and the SOFTWARE ZEN 2.3 Lite. Leaf samples were imaged at 20×, 100×and 500× magnifications (Figure 6c). The number of stomata was counted in a selected area of the image to calculate Stomatal Density (SD; n mm^−2^), inter-stomatal distance (LD), and stomatal aperture size (semi-major axes S-mA). 

In addition, the micromorphology of both the adaxial and abaxial leaf surfaces was examined by scanning electron microscopy (SEM) for the selected five cultivars (cvs. Malvasia del Lazio, Sangiovese, Cabernet Sauvignon, Maturano and Barbera). From each leaf sample, two square sections (8 × 8 mm) were excised from the basal and apical regions and mounted on aluminum stubs. Samples were dehydrated and dried using the critical-point drying method with CO_2_ (Model CPD-020, Balzers Union, Liechtenstein), then sputter-coated with gold using a Balzers MED 010 unit (Balzers Union Ltd., Balzers, Liechtenstein). Observations and micrographs were obtained with a JEOL JSM-5200 scanning electron microscope (JEOL Ltd., Tokyo, Japan). Five images were captured for texture measurements on both the adaxial and abaxial surfaces at 100× and 500× magnification. Images obtained at the two magnifications were analysed separately. Within each magnification, accelerating voltage, working distance, detector, image resolution, brightness and contrast settings were kept constant for all samples.

Image analysis was performed using ImageJ software bundled with Java 8 (National Institutes of Health, Bethesda, MD, USA) [54]. Image-based surface texture was characterized using the SurfCharJ plugin [55]. Conventional SEM grayscale intensity is not a direct measurement of surface height. Because no calibrated conversion between pixel intensity and the vertical coordinate was available, the analysis provided image-texture descriptors derived from grayscale intensity in arbitrary units (a.u.). SurfCharJ was used to calculate comparative descriptors of grayscale variation, hereafter referred to as image-based surface-texture parameters. This plugin calculates roughness-related parameters from grayscale images of the leaf surface: Ra (arithmetic mean roughness) and other roughness parameters, such as Rv (maximum valley depth), Rp (maximum peak height) and Rq (root mean square roughness of the surface profile) (Figure 6e). 

### 4.4. Ecophysiological Proxies

Ecophysiological proxies were assessed on the same selected cultivars to evaluate plant functional responses in the urban environment. The monitored parameters included leaf chlorophyll content (CHL—μmol∙m^−2^) and leaf diffuse reflectance, which allowed the calculation of ecophysiological indices. Measurements were conducted before leaf sampling for digital phenotyping and the quantification of leaf-intercepted PM. In particular, leaf chlorophyll content was measured non-destructively using a hand-held MC-100 Chlorophyll Concentration Meter (Apogee Instruments. Inc., Logan, UT, USA) [23]. Measurements were taken on five leaves per cultivar, on the leaf blade, avoiding the main veins and damaged areas, to ensure reliable readings. The diffuse reflectance (spectral signature) for each leaf was measured using a portable, high-resolution spectrophotometer (ASD FieldSpec^®^3. Analytical Spectral Devices, Boulder, CO, USA) that recorded the full range of the solar irradiance spectrum (350–2500 nm). Three hyperspectral measurements were made on each leaf, with 15 internal scans per measurement. Reflectance measurements were used to derive vegetation indices (VIs) related to plant physiological status: (i) the Photochemical Reflectance Index (PRI; (R531 − R570)/(R531 + R570)), which indicates the efficiency with which vegetation uses incident light for photosynthesis [56]; (ii) the Water Band Index (WBI; R900/R970), which is sensitive to leaf water content; and (iii) the Normalized Difference Infrared Index (NDII; (R819 − R1649)/(R819 + R1649)) [57], using the contrast between near-infrared reflectance, mainly influenced by leaf internal structure, and short-wave infrared reflectance, which is strongly affected by water absorption. These indices were used as proxies for Equivalent Water Thickness (EWT), which is an early indicator of plant dehydration and drought stress, with higher values generally associated with higher water content (1) and lower values indicating possible water limitation or stress (−1).

### 4.5. Particulate Density Analysis

Leaf-surface PM deposition was quantified through scanning electron microscopy (SEM) imaging and subsequent particle counting analysis. Samples were analysed using a Phenom ProX SEM (Phenom-World BV, Eindhoven, The Netherlands). A charge-reducing sample holder specifically designed for biological specimens was employed. The SEM was operated in backscattered electron (BSE) mode at 5 keV accelerating voltage to reduce surface charging, following procedures adapted from Baldacchini et al. [58] and Sgrigna et al. [59]. Leaf fragments, derived from three leaves of the five collected, were placed in the holder using double-sided carbon conductive PELCO Tabs (Ted Pella, Inc., Redding, CA, USA). Particles were analysed directly on leaf surfaces, without transferring them to filters. For each sampled leaf, 20 SEM images were acquired: 10 from the adaxial (upper) surface and 10 from the abaxial (lower) surface, resulting in 60 images per cultivar. Each image covered a scan area of approximately 150 × 150 μm, at a resolution of 1024 × 1024 pixels. Images were selected randomly across the leaf surface to avoid operator bias. SEM images were processed using Gwyddion [60], applying a color threshold-based grain analysis to identify individual particles and extract their equivalent circular diameter (deq). Particles smaller than the pixel resolution threshold (~0.3 μm in diameter) were excluded, as well as particles >10 μm, which accounted for <0.1% of total detections. The analyzed particle set therefore corresponds to PM 0.3–10. For descriptive analysis, particles were classified into three size classes, based on particle diameter: PM 0.3–1; PM 1–2.5; and PM 2.5–10. Particle density per unit leaf surface (expressed as number of particles per square millimeter—PM mm^−2^) was calculated by normalizing the total number of detected particles to the cumulative image area analyzed for each replicate. The resulting values were averaged across replicates, and standard error (SE) was computed to represent variability among leaves. PM accumulated on the adaxial and abaxial leaf surfaces was quantified and characterized using scanning electron microscopy coupled with energy-dispersive X-ray spectroscopy (SEM) with a Phenom ProX system. Three particle-size classes were considered: PM 0.3–1, corresponding to particles having aerodynamic diameter ≤ 1 µm and defined as “ultrafine particles”; PM 1–2.5, corresponding to particles having aerodynamic diameter ≤ 2.5 µm and defined as “fine particles”; and PM 2.5–10, corresponding to particles having aerodynamic diameters > 2.5 and ≤10 µm and defined as “coarse particles”. The two leaf surfaces were analysed separately to assess differences in particle deposition between the adaxial and abaxial sides.

### 4.6. Data Analysis

Cultivar-based differences were analysed in terms of leaf morphological and anatomical traits, leaf PM accumulation, and ecophysiological parameters, including chlorophyll content and vegetation indices. For each cultivar, ten leaves were collected from the two available vines using a stratified random sampling procedure. Sampling was designed to obtain a representative set of leaves with respect to leaf age, position within the canopy, exposure, and height above ground. The ten leaves were subsequently allocated to two non-overlapping analytical subsets: five leaves were used for leaf morphological and anatomical analyses, whereas the remaining five leaves were used for PM analysis. Consequently, each analytical dataset comprised five leaf-level observational units per cultivar. Images, microscopic fields, particle-size classes, leaf surfaces, and repeated instrumental measurements obtained from the same leaf were treated as technical or repeated subsamples and were averaged or aggregated at the leaf level before statistical analysis. Before the statistical analysis, the dataset was screened for missing values, recording errors, implausible measurements, and influential observations. Cultivar differences were evaluated using the non-parametric Kruskal–Wallis test, with grapevine cultivar as the categorical factor. Statistical significance was assessed at *p* ≤ 0.05. When a significant cultivar effect was detected, pairwise comparisons among cultivars were performed using Dunn’s post-hoc test, with correction for multiple comparisons.

In addition, relationships among the measured variables were investigated by calculating Spearman rank correlation matrix. The matrix was calculated from the five cultivar-level means because the leaves used for PM analysis differed from those used for morphological and anatomical characterisation; correlations between these measurement domains were calculated using cultivar means rather than paired leaf-level observations (n = 5 per coefficient). Therefore, the coefficients describe associations among cultivar averages. Before correlation analysis, variables were screened for conceptual redundancy based on their biological interpretation and measurement meaning. When two variables represented the same derived information, the variable providing the most direct or biologically interpretable measurement was retained. However, no formal multivariable collinearity analysis was performed because the correlation dataset contained only five cultivar-level observations. Spearman’s rank correlation coefficient (ρ) was used to quantify the direction and strength of monotonic associations between leaf traits, PM accumulation, surface image-based surface texture descriptors (roughness), chlorophyll-related parameters, and vegetation indices. Correlation coefficients ranged from −1 to +1, with values close to ±1 indicating strong relationships and values close to 0 indicating weak or absent associations. All statistical analyses were performed using XLSTAT 2016.02.2845.

## Figures and Tables

**Table 1:** Thermal bioclimatic indices of the urban area hosting the vineyard.

Indicator	Unit	2025 Value	2009–2024 Mean	2025 Difference	Potential Implication for Grapevine
Winkler Index (WI)	GDD	2703	≈2160	+25.1%	Acceleration of phenological development, altered berry ripening, increased heat stress
Temperature during ripening—CI (Cool night index)	°C	18.8	22.99	−4.19 °C	Improved conditions for berry quality at the end of a hot growing season
Growing-season temperature (GST)	°C	22.69	22.48	+0.21 °C	Acceleration of phenological development, increased exposure to multiple stresses
Hot Days—days with temperature exceeding 30 °C	days	143	≈84	+70.0%	Increased stress affecting vine physiology and berry quality

**Table 2:** Mean values of vegetation indices measured by spectroradiometer in the investigated grapevine varieties. WI: Water Index; PRI: Photochemical Reflectance Index; NDII: Normalized Difference Infrared Index. Different lowercase letters within each column indicate statistically significant differences among varieties according to post hoc test; (ns) non-significant differences.

Cultivar	WI	*Sign.*	PRI	*Sign.*	NDII	*Sign.*
Malvasia del Lazio	1.030 ± 0.005	*ab*	0.027 ± 0.005	*ns*	0.466 ± 0.005	*b*
Sangiovese	1.050 ± 0.001	*b*	0.029 ± 0.001	*ns*	0.439 ± 0.000	*ab*
Cabernet Sauvignon	1.024 ± 0.001	*a*	0.027 ± 0.002	*ns*	0.442 ± 0.000	*ab*
Maturano	1.033 ± 0.001	*ab*	0.031 ± 0.000	*ns*	0.44 ± 0.000	*ab*
Barbera	1.038 ± 0.000	*ab*	0.038 ± 0.001	*ns*	0.416 ± 0.001	*a*

**Table 3:** Leaf morphological traits (area, perimeter and aspect ratio) and stomatal traits (density, linear distance and semi-major axes) for the selected cultivars under urban growing conditions. Different lowercase letters within each column indicate statistically significant differences among varieties according to post hoc test; (ns) non-significant differences.

Cultivar	Leaf Area (cm^2^)	*Sign.*	Leaf Perimeter (cm)	*Sign.*	Aspect Ratio (L/W)	*Sign.*
Malvasia del Lazio	240.5 ± 17.3	*ab*	124.5 ± 8.1	*ab*	1.02 ± 0.04	*ns*
Sangiovese	252.3 ± 53.3	*ab*	156.6 ± 22.8	*b*	0.93 ± 0.1	*ns*
Cabernet Sauvignon	151.3 ± 21.8	*a*	88.9 ± 6.2	*a*	0.89 ± 0.1	*ns*
Maturano	302.8 ± 25.5	*b*	118.0 ± 5.9	*ab*	0.96 ± 0.1	*ns*
Barbera	229.4 ± 39.3	*ab*	102.0 ± 5.5	*ab*	0.90 ± 0.02	*ns*
**Cultivar**	**Stomata Density (SD) (n·mm^−2^)**	***Sign.***	**Linear** **Distance** **(µm)**	***Sign.***	**Semi-Major Axes** **(µm)**	***Sign.***
Malvasia del Lazio	320.9 ± 81.0	*ns*	30.5 ± 1.6	*ab*	15.3 ± 0.8	*ab*
Sangiovese	192.3 ± 44.7	*ns*	30.2 ± 2.8	*a*	15.1 ± 1.4	*a*
Cabernet Sauvignon	156.1 ± 68.3	*ns*	34.6 ± 0.9	*b*	17.3 ± 0.4	*b*
Maturano	244.8 ± 66.0	*ns*	33.9 ± 2.3	*ab*	12.8 ± 1.2	*a*
Barbera	143.2 ± 16.5	*ns*	32.9 ± 0.5	*ab*	16.4 ± 0.2	*ab*

**Table 4:** SEM-derived image-texture parameters of the abaxial and adaxial leaf surfaces of five grapevine cultivars. Values (a.u.) represent comparative image-texture descriptors, conventionally described as: Rq, root mean square roughness; Ra, arithmetic mean roughness; Rv, maximum valley depth; Rp, maximum peak height; and Rt, total height of the roughness profile. The descriptors reflect comparative variation in SEM grayscale intensity and do not represent measurements of physical surface height or depth. Different letters within each column indicate statistically significant differences among cultivars according to the post hoc test (*p* ≤ 0.05), (ns) non-significant differences.

Cultivar Abaxial Surface	Rq		Ra		Rv		Rp		Rt	
			(a.u.)							
Malvasia del Lazio	286.92	*b*	220.55	*b*	−615.91	*ns*	1093.92	*ns*	1709.83	*ns*
Sangiovese	215.01	*a*	165.94	*a*	−664.80	*ns*	879.25	*ns*	1544.05	*ns*
Cabernet Sauvignon	239.99	*ab*	183.27	*ab*	−657.72	*ns*	999.87	*ns*	1657.59	*ns*
Maturano	218.05	*a*	163.32	*a*	−692.46	*ns*	1026.45	*ns*	1718.91	*ns*
Barbera	224.38	*ab*	158.43	*a*	−699.70	*ns*	1316.95	*ns*	2016.65	*ns*
**Adaxial Surface**	**Rq**		**Ra**		**Rv**		**Rp**		**Rt**	
			(a.u.)							
Malvasia del Lazio	245.81	*ab*	197.54	*b*	−619.22	*b*	780.30	*a*	1399.52	*a*
Sangiovese	267.47	*b*	207.64	*b*	−815.58	*a*	1163.67	*c*	1979.24	*c*
Cabernet Sauvignon	235.13	*ab*	184.94	*ab*	−671.50	*ab*	896.23	*abc*	1567.73	*abc*
Maturano	195.93	*a*	153.39	*a*	−579.59	*b*	724.66	*ab*	1304.25	*ab*
Barbera	273.93	*ab*	201.81	*ab*	−1026.21	*a*	1233.29	*bc*	2259.50	*bc*
**Cultivar** **Abaxial Surface**	**Rq**		**Ra**		**Rv**		**Rp**		**Rt**	
			(a.u.)							
Malvasia del Lazio	286.92	*b*	220.55	*b*	−615.91	*ns*	1093.92	*ns*	1709.83	*ns*
Sangiovese	215.01	*a*	165.94	*a*	−664.80	*ns*	879.25	*ns*	1544.05	*ns*
Cabernet Sauvignon	239.99	*ab*	183.27	*ab*	−657.72	*ns*	999.87	*ns*	1657.59	*ns*
Maturano	218.05	*a*	163.32	*a*	−692.46	*ns*	1026.45	*ns*	1718.91	*ns*
Barbera	224.38	*ab*	158.43	*a*	−699.70	*ns*	1316.95	*ns*	2016.65	*ns*
**Adaxial Surface**	**Rq**		**Ra**		**Rv**		**Rp**		**Rt**	
			(a.u.)							
Malvasia del Lazio	245.81	*ab*	197.54	*b*	−619.22	*b*	780.30	*a*	1399.52	*a*
Sangiovese	267.47	*b*	207.64	*b*	−815.58	*a*	1163.67	*c*	1979.24	*c*
Cabernet Sauvignon	235.13	*ab*	184.94	*ab*	−671.50	*ab*	896.23	*abc*	1567.73	*abc*
Maturano	195.93	*a*	153.39	*a*	−579.59	*b*	724.66	*ab*	1304.25	*ab*
Barbera	273.93	*ab*	201.81	*ab*	−1026.21	*a*	1233.29	*bc*	2259.50	*bc*

**Table 5:** Spearman correlation matrix among morphological traits—leaf area (LA—cm^2^) and perimeter (LP—cm), stomatal density (SD—n·mm^−2^) and their semi-major axes (S-mA—µm), normalized Difference Infrared Index (NDII), total particulate matter (PM TOT—number of particles per mm^2^) accumulated on abaxial (AB) and adaxial (AD) leaf surfaces. Values represent image-based texture descriptors: arithmetic mean roughness (Ra), maximum valley depth (Rv), maximum peak height (Rp) for abaxial (AB) and adaxial (AD) leaf surfaces. Blue cells indicate positive correlations, whereas red cells indicate negative correlations.

	LA	LP	SD	S-mA	NDII	PM TOT AB	PM TOT AD	AB-Ra	AB-Rv	AB-Rp	AD-Ra	AD-Rv	AD-Rp
LA	1.00												
LP	0.65	1.00											
SD	0.44	0.66	1.00										
S-mA	−0.88	−0.73	−0.54	1.00									
NDII	0.14	0.33	0.64	−0.32	1.00								
PM TOT AB	−0.09	0.12	0.43	−0.18	0.44	1.00							
PM TOT AD	−0.09	−0.15	0.36	−0.04	0.12	0.72	1.00						
AB-Ra	−0.23	0.03	0.60	0.16	0.78	0.25	0.22	1.00					
AB-Rv	−0.39	−0.01	−0.07	0.27	0.04	0.54	0.05	−0.02	1.00				
AB-Rp	−0.01	−0.01	0.39	0.05	0.29	−0.30	0.17	0.63	−0.71	1.00			
AD-Ra	−0.31	0.16	0.21	0.27	0.27	−0.28	−0.10	0.58	−0.27	0.73	1.00		
AD-Rv	0.15	−0.14	0.22	−0.13	0.33	0.61	0.39	0.17	0.47	−0.38	−0.61	1.00	
AD-Rp	−0.26	−0.02	−0.38	0.20	−0.36	−0.58	−0.37	−0.23	−0.47	0.32	0.50	−0.94	1.00

## Data Availability

The original contributions presented in this study are included in the article/Supplementary Materials. Further inquiries can be directed to the corresponding authors.

## Supplementary Materials

The following supporting information can be downloaded at: https://www.mdpi.com/article/10.3390/plants15193014/s1, Table S1: Bioclimatic indices used for the climatic characterization of the area of Rome close to the urban vineyard (city center) (Source: Biasi et al., 2019) [26]; Table S2: Daily particulate matter concentrations (PM2.5 and PM10) in the area of Rome close to the urban vineyard during the days prior to leaf sampling for analysis of particulate matter retention on leaf abaxial and adaxial surfaces.
