# Supplementary material for: Plant–Atmosphere Interactions in Urban Vineyards of Rome: A Digital Phenotyping Approach for the Functional Selection of Grapevine Cultivars
Source: Plants (Basel). 2026 Sep 30;15(19):3014. doi: 10.3390/plants15193014 (PMC13667060; doi:10.3390/plants15193014)
Supplement: Supplementary file 1 [file plants-15-03014-s001.zip › plants-4508302-supplementary.pdf]

Supplementary materials

Table S1. Bioclimatic indices used for the climatic characterization of the area of Rome close to the urban vineyard (city center) (Source: Biasi et al., 2019) [26].

Bioclimatic index	Abbreviation	Formula	Unit
Winkler Index	WI	WI = Σ (T_m − 10 °C)<br>1 Apr–31 Oct	degree-days<br>(DD)
Cool Night Index	CI	CI = T_{min} (September)	°C
Growing Season Temperature	GST	GST = mean T (April–October)	°C
Hot Days	HDs	days with temperature exceeding 30 °C	(n)

Table S2. Daily particulate matter concentrations (PM2.5 and PM10) in the area of Rome close to the urban vineyard during the days prior to leaf sampling for analysis of particulate matter retention on leaf abaxial and adaxial blade.

Date	PM10 μg m^{-3}	PM2.5 μg m^{-3}
2025-06-19	25	13
2025-06-20	31	16
2025-06-21	27	15
2025-06-22	24	14
2025-06-23	25	15
2025-06-24	25	14
2025-06-25	27	16
2025-06-26	29	15
2025-06-27	35	15
2025-06-28	21	11
2025-06-30	27	19
2025-07-01	27	18
2025-07-02	27	16
2025-07-03	27	16
2025-07-04	29	19
2025-07-05	23	16
2025-07-06	18	14
2025-07-07	22	8
2025-07-08	28	9
